# Triboluminescence of metal halide perovskite films

**DOI:** 10.1038/s41377-025-02032-4

**Published:** 2025-11-06

**Authors:** Hao Tian, Fengke Sun, Jun Chen, Fusai Sun, Xiaotao Liu, Yang Li, Zhanjun Zhu, Junxue Guo, Bo Zhou, Xin Guo, Can Li

**Affiliations:** 1 State Key Laboratory of Photoelectric Conversion and Utilization of Solar Energy, Dalian Institute of Chemical Physics, Chinese Academy of Sciences, Dalian, China; 2 University of Chinese Academy of Sciences, Beijing, China; 3 State Key Laboratory of Molecular Reaction Dynamics, Dalian Institute of Chemical Physics, Chinese Academy of Sciences, Dalian, China

**Keywords:** Optical materials and structures, Other photonics

## Abstract

Metal halide perovskites (MHPs) have shown fantastic properties such as photoelectric conversion, electroluminescence, light amplification, ferroelectricity, and flexoelectricity, becoming versatile materials in diverse fields. Herein, we report a previously undiscovered nature of the MHP film, i.e. triboluminescence (TL), and elucidate its underlying mechanism. The TL response is triggered when an MHP film is scraped by specific materials (like Cu, Au, Pt) with a lower Fermi level than that of the MHP. The friction induces electron transfer from MHP to scraper, generating a positive electrostatic field on the surface of the MHP film, which leads to stretched MHP lattices owing to electrostatic repulsion, consequently enhancing fluorescence and charge carrier lifetime. If the friction material (like Al) has a higher Fermi level, electron transfer process, electrostatic field direction, and lattice structure change are just the opposite, resulting in reduced fluorescence. This work unveils the TL phenomenon of the MHP film and the unique mechanism, opening up new avenues for explorations on the appealing MHPs.

## Abstract (web summary)

This study reveals the triboluminescence (TL) phenomenon of the metal halide perovskite film for the first time, which is based on a new mechanism of friction-induced lattice stretching, significantly distinct from that of the conventional TL.

## Graphical abstract

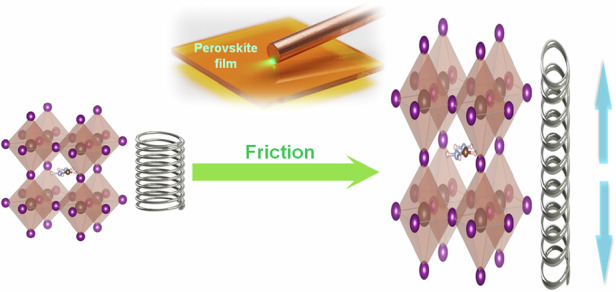

## Introduction

Metal halide perovskites (MHPs, ABX_3_) with remarkable optoelectronic properties have emerged as highly promising materials in diverse fields, including solar cells, light-emitting diodes, photoelectric detectors, etc^1–3^. Apart from intrinsic optoelectronic characteristics, MHPs also present unique ferroic properties such as ferroelectricity, piezoelectricity, ferroelasticity, and flexoelectricity^4–7^. These properties are subject to external mechanical stresses which cause changes in microstructures of MHPs, thus motivating noticeable electrical responses. The optical responses of MHPs to mechanical stresses have, however, received less attention.

The optical responses of a material to external stresses can be classified as a kind of triboluminescence (TL), also known as mechanoluminescence. Since the first observation of the TL from scraping hard sugars in 1605^8^, various TL materials and corresponding luminescence mechanisms have been reported, including sucrose and tartaric acid (fracture-induced gas excitation)^9–11^, doped-semiconductor materials (stress-induced electrons de-trapping)^12–15^, piezoelectric crystals (piezoelectric discharge)^16–19^, and organic materials (molecular aggregation)^20,21^. The TL phenomenon has also been occasionally mentioned for perovskite-type materials such as traditional oxide perovskites (e.g. Sr_n+1_Sn_n_O_3n+1_:Sm^3+^, BaTiO_3_–CaTiO_3_, MgGeO_3_:Mn^2+^, and LiTaO_3_:Pr^3+^) based on the electrons de-trapping mechanism, an antiperovskite ([(CH_3_)_3_NH]_3_(MnCl_3_)[MnCl_4_]) stemming from the brittle fracture of its crystal, and a low-dimensional perovskite crystal ([C_19_H_18_P]_2_MnBr_4_) originated from the piezoelectric discharge^12,15,17,22–24^. However, the TL of such perovskite-type materials can only be triggered by mechanical stresses in the form of single crystals. For the MHPs being in the limelight in the field of optoelectronics, their TL has never been documented, particularly in the polycrystalline film state.

On the other hand, these traditional TLs are generated from the materials deformation induced by various mechanical stresses such as bending, collapsing, squeezing, and friction. It is noteworthy that the friction is quite different from others because it can not only cause the deformation but also result in the interfacial charge transfer between two objects^25,26^. Such friction-induced charge transfer can alter electrical behaviors of materials^27–29^, but its effect on optical properties, in particular on the TL response, has never been investigated.

In this study, we report the TL of the MHP films based on a friction-induced charge transfer mechanism. We find that the MAPbI_3_, MAPbBr_3_, FAPbBr_3_, and CsPbBr_3_ films exhibit significantly enhanced and hypsochromic photoluminescence (PL) when being scraped on the surface, whereas their PL behaviors are not changed by any mechanical stress-induced deformations. More interestingly, when being used to scrape the perovskite films, only materials with lower Fermi levels than those of the perovskites can enhance the PL intensity of MHPs, triggering the TL, while other materials with higher Fermi levels reduce the PL intensity of MHPs. We demonstrate that during the friction process, electrons transfer from the surface of the perovskite film to the scraper with a lower Fermi level, driven by the Fermi-level difference, which induces the accumulation of positive charges on the surface of the perovskite film. The resultant positive electrostatic field stretches the surface lattice structure of the perovskite film containing positively charged defects due to the electrostatic repulsion, leading to reduced trap density and a wider bandgap, which contribute to the enhanced and hypsochromic PL emission. To the best of our knowledge, this is the first report on the TL of MHPs, following a unique TL mechanism.

## Results

**Fig. 1:**
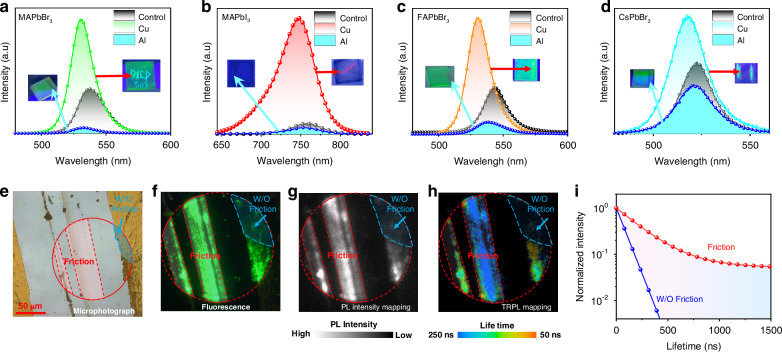
**Friction-induced photoluminescence changes of the MHP film**. PL spectra of **a** MAPbBr_3_, **b** MAPbI_3_, **c** FAPbBr_3_, and **d** CsPbBr_3_ films scraped by Cu and Al. **e** Microphotograph, **f** fluorescence microphotograph, **g** confocal PL mapping (1 μW laser), **h** TRPL mapping images and **i** fitting curves of the Cu-scraped MAPbBr_3_ film; red and blue dash lines indicate areas with and without friction, respectively

The TL was observed when scraping the MAPbBr_3_ film with a metal (e.g., a copper (Cu) rod), as recorded in the Supplementary Video 1. The pristine MAPbBr_3_ film exhibits green fluorescence under the UV illumination. After scraping, the fluorescence becomes brighter and stronger, which can be observed with the naked eyes. The PL spectra (Fig. 1a) display that the friction area shows an obviously stronger fluorescence band accompanying with a blue-shift of 10 nm compared to the control (friction-free) area, which is a typical TL response. Then, we investigate the universality of this finding by using other kinds of perovskite films and friction materials. We find that the similar blue-shift and enhancement in the PL spectra to those of the MAPbBr_3_ film are also observed when scraping the MAPbI_3_, FAPbBr_3_, and CsPbBr_3_ films with the Cu rod (Fig. 1b–d), indicating that the TL phenomenon is pervasive for various perovskites. Very interestingly, by scraping the MAPbBr_3_ film with materials like sliver (Ag), gold (Au), platinum (Pt), and stainless steel, the TL can still be generated; however, other materials such as aluminum (Al), plastic and glass do not initiate the TL response, but remarkably weaken the fluorescence with a subtle blue-shift.

To further confirm the TL, the confocal laser scanning microscope (CLSM) was measured for the Cu-scraped MAPbBr_3_ film. Figure 1e displays the photograph of the Cu-friction area and the control area without friction, as marked by a red circle. It can be clearly seen that in the fluorescence microscopy (Fig. 1f), the Cu-friction area is much brighter than the control one. The CLSM mapping results further show a pronounced increase in fluorescence intensity within the friction area (Fig. 1g). Furthermore, the time-resolved PL (TRPL) spectroscopy reveals that the friction area exhibits a significantly prolonged carrier lifetime relative to the control region (Fig. 1h, i). Impressively, the enhanced fluorescence of the Cu-scraped MAPbBr_3_ film lasts for a long time with a high fluorescence intensity (Supplementary Fig. S1), very different from the TL of traditional material systems that display instantaneous light emission. It is noteworthy that such changes in PL properties cannot be observed by bending or compressing the MHP film (Supplementary Fig. S2), which is in agreement with a previous finding^30^. This observation is also distinct from traditional TL materials which optically respond to the mechanical force-induced deformation.

**Fig. 2:**
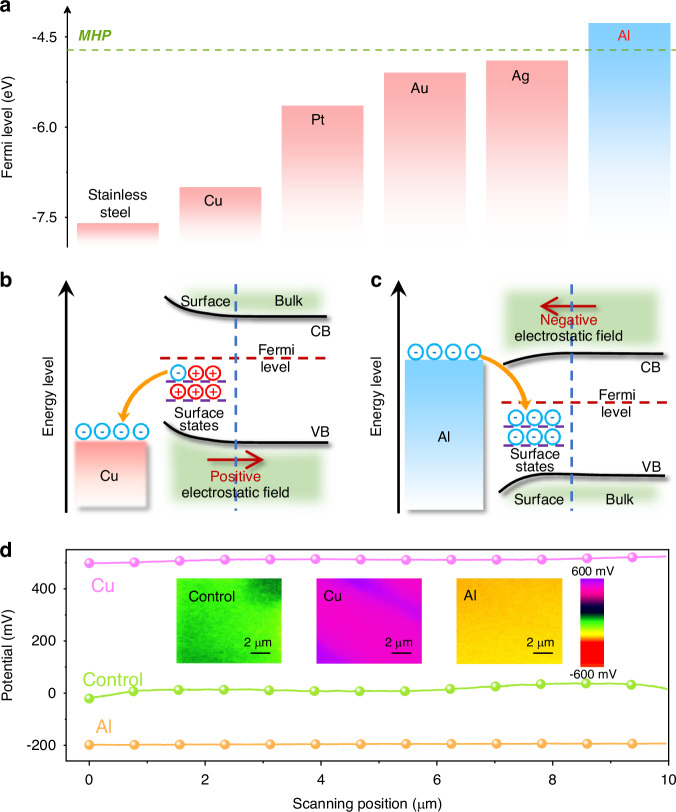
**Fermi-level difference-induced charge transfer and surface electrostatic filed of MHP films**. **a** Fermi-level alignment of MHPs and metals used for scraping the perovskite film. Schematic diagram of the friction-induced charge transfer between MHP and materials with **b** high and **c** low Fermi levels. **d** Surface potential distribution of control, Cu-scraped and Al-scraped regions (10 μm × 10 μm) in the MAPbBr_3_ film; inset: surface potential mapping

To figure out the underlying reason for friction-induced changes in PL properties, we pay attention to the Fermi levels of these perovskites and friction materials. It is known that the friction process can lead to an increased overlap in local electron clouds between the two interacting materials promoting the interfacial electron transfer, which is influenced by their Fermi levels^28^. The ultraviolet photoelectron spectroscopy (UPS) measurement (Supplementary Fig. S3) presents that Fermi levels of these perovskites locate around −4.7 eV, higher than those of the metals which can induce the TL, but lower than that of Al, as shown in Fig. 2a. These results suggest that the TL highly depends on the relative Fermi-level positions of perovskites and friction materials. When two materials are rubbed against each other, the difference in their Fermi levels drives the transfer of surface-state electrons from the material with a higher Fermi level to the material with a lower one, resulting in the development of static electricity on the frictional surfaces of both materials^27^. In our case, the metals like Cu, Ag, etc. have lower Fermi levels than that of perovskites, accepting surface electrons from perovskites, which leads to a positive electrostatic field on the surface of the perovskite film (Fig. 2b). Conversely, other materials like Al with higher Fermi levels than that of perovskites will result in a negative electrostatic field on the perovskite surface after friction (Fig. 2c).

**Fig. 3:**
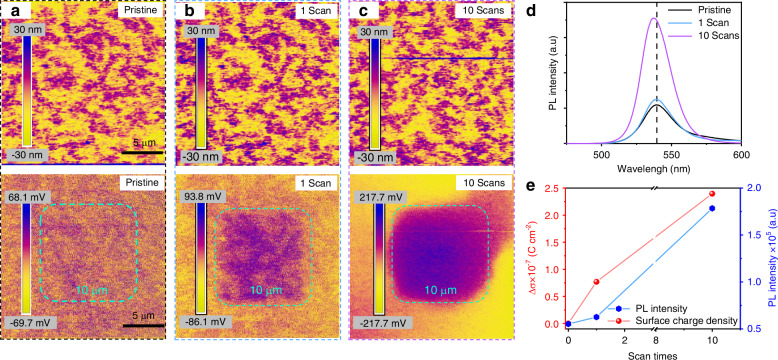
**In situ monitoring of the enhanced surface potential of the MHP film using the KPFM probe as a scraper**. Surface potential distributions of the MAPbBr_3_ film (20 μm × 20 μm) with different scanning times within a 10 μm × 10 μm area (marked by green dash lines): **a** pristine, **b** 1 scan, and **c** 10 scans; Top: height diagram and bottom: surface potential mapping, respectively. **d** PL spectra of MAPbBr_3_ films scanned by 0, 1, and 10 times, respectively, using the KPFM probe. **e** Correlation between changes in the surface charge density and the PL intensity and scan times using the KPFM probe

In order to prove the generation of electrostatic fields on the surface of the perovskite film, we used the Kelvin Probe Force Microscopy (KPFM) to evaluate the differences in the surface potential distribution of Cu-, Al-scraped and control samples. To avoid experimental errors caused by sample variations, each perovskite film was divided into three regions; one of them was not scraped as the control, and the other two were scraped by Cu and Al, respectively, with the same friction way (see more details in Supplementary Fig. S4). Figure 2d depicts the lateral distribution of the surface potential (Supplementary Fig. S5), revealing that the control region shows a surface potential of ~20 mV, while the Cu- and Al-scraped ones display a positive value of 500 mV and a negative value of −200 mV, respectively. Furthermore, we utilized the platinum (Pt)-coated Ir probe equipped in the KPFM as a scraper to repetitively scan the MAPbBr_3_ film surface under non-biased conditions, simulating the friction process to in situ monitor the charge transfer. Since the probe has a lower Fermi level than that of perovskite, positively enhanced surface potentials can be anticipated. The scan was conducted in a 10 μm × 10 μm area, and the surface potential signal was acquired in a 20 μm × 20 μm area, as shown in Fig. 3. The height diagram indicates that the surface morphology of the perovskite film keeps unchanged during the scanning process. Interestingly, in the potential map (Supplementary Fig. S6), the scanned area shows a higher surface potential than other areas after the first scan, presenting a small shoulder peak at ~115 mV in the contact potential difference (CPD) distribution diagram, which is obviously higher than ~70 mV for the pristine film. After 10 scans, the surface potential of the scanned area significantly increases, with an additional peak appeared at ~210 mV. Moreover, the change in the surface charge density, induced during the KPFM probe simulation process, was calculated using the one-dimensional Maxwell equation: ${\rm{\sigma }}={\varepsilon }_{0}\varepsilon \varDelta {V}_{{CPD}}/d$ (σ: charge density; *ε*_0_, *ε*: vacuum and perovskite dielectric permittivity, respectively; *∆V*_*CPD*_: the difference in CPD; d: the effective distance between the perovskite film and the probe)^30,31^. The results indicate that the surface charge density of the perovskite film is increased by 7.7×10^−8^ C cm^−2^ after one scan, and further by$\,2.4\times {10}^{-7}$ C cm^−2^ after 10 scans. The PL spectra of the perovskite films with different KPFM probe scanning times are then measured. Figure 3d demonstrates progressive enhancements in the PL intensity with repeated scans, accompanied by a slight blue-shift of the emission maximum. The correlation between the increased surface charge density and PL intensity and the KPFM probe scanning times is established, as shown in Fig. 3e, which can quantitatively reflect the charge transfer process and the PL enhancement. These results strongly support that the friction by Cu or Pt with a low Fermi level induces the accumulation of positive charges forming a positive electrostatic field on the surface of the perovskite film; on the contrary, the friction by Al with a high Fermi level introduces a negative electrostatic field.

**Fig. 4:**
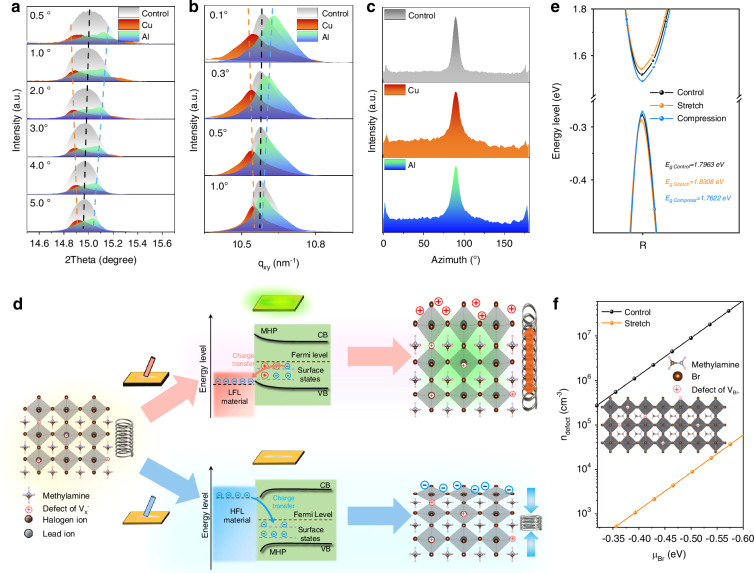
**Crystal lattice structure changes, resulted from the friction-induced surface electrostatic field of the MHP film**. **a** Diffraction peaks of (100) crystal facets in GIXRD patterns. **b** One-dimensional integration plots of GIWAXS tested at different incident angles for control, Cu- and Al-scraped regions on the surface of the MAPbBr_3_ film. **c** Crystal plane orientation of the MAPbBr_3_ film after friction with Cu and Al. **d** Schematic diagrams of stretched and compressed perovskite lattice structures caused by friction with low Fermi-level (LFL) and high Fermi-level (HFL) materials. **e** Band structures obtained from DFT calculations. **f** Defect density of the perovskite film before and after the lattice stretch

Next, we investigate the effect of the friction-induced electrostatic field on the surface of the MAPbBr_3_ film on the crystal lattice structure by the grazing incident X-ray diffraction (GIXRD). The samples for this measurement were prepared by the same way as that for the KPFM, containing three regions (control, Cu friction and Al friction) in the MAPbBr_3_ film, which were detected using the GIXRD with different incident angles (0.5-5°). The results are provided in Supplementary Fig. S7 and diffraction signals of (100) crystal facets of the three regions are extracted and presented in Fig. 4a. Compared to the control region with the (100) diffraction peak at 14.9°, Cu- and Al-scraped ones exhibit shifted (100) signals to a lower and higher diffraction angle, respectively. It has been known that the peak shift to a lower angle signifies a stretched lattice, while that to a higher angle indicates a compressed lattice^32–36^. As the incident angle increases from 0.5 to 5° corresponding to the increased detecting depth, such shifts to both directions become gradually smaller, suggesting that the lattice structure changes more obviously on the film surface than that in the bulk. We further conducted the grazing incidence wide-angle X-ray scattering (GIWAXS) measurements (Supplementary Fig. S8), and the one-dimensional integration plots (Fig. 4b) exhibit similar results in the lattice change without varying crystal orientations (Fig. 4c). These results suggest that the friction-induced positive (by Cu) and negative (by Al) electrostatic fields on the surface of the perovskite film lead to stretched and compressed perovskite lattices, respectively. The changed lattice structures can be explained by the interaction between the type of the electrostatic field and the surface charge state of the perovskite film. It has been reported that positively charged undercoordinated Pb^2+^ defects are abundant on the surface of the perovskite film due to the migration of halide ions in [PbX_6_]^4-^ octahedra^37,38^. Upon the friction by Cu, the generated positive electrostatic field forms the electrostatic repulsion interaction with the positively charged surface, leading to the stretch of the perovskite lattice. On the contrary, the Al friction-induced negative electrostatic field causes the lattice compression due to the coulomb attraction (Fig. 4d).

Theoretical studies in previous works have demonstrated that the changed lattice structure may influence bandgap and defect state of the perovskite^35,36,39–42^. Our results obtained from the density functional theory (DFT) calculations also indicate that the Cu friction-induced lattice stretch of MAPbBr_3_ widens its bandgap (Fig. 4e, Supplementary Fig. S9 and Supplementary Table S1), which is consistent with the blue-shifted emission band in the PL spectra, as discussed above. The calculated defect energies and density (Fig. 4f) using Defect Energy Calculation (DEC) and Defect Density Calculation (DDC) modules of Defect^43,44^ exhibit a three-order-of-magnitude reduction in defect density for the stretched MAPbBr_3_ relative to the control within the identical chemical environment. The reduced defect density suggests a lower non-radiative recombination rate, which improves the PL intensity and prolongs the carrier lifetime of MHPs. For the case of the Al friction-induced lattice compression, the narrowed bandgap and increased defect density are observed from the DFT calculations. The narrowed bandgap is supposed to make the fluorescence band slightly red-shifted; however, a subtle blue-shift is observed in the PL spectrum of the Al-scraped film as mentioned above, which is most likely due to the synergistic effect of the lowered bandgap and the reduced film thickness (see details in Supplementary Fig. S10)^45,46^. These calculations demonstrate that the TL of the perovskite film stems from the stretched lattice structure.

According to these results, we propose the following mechanism for the TL of the MHP films (Fig. 4d). The friction process improves the overlap of electron clouds of the friction material and the MHP, resulting in interfacial charge transfer. When the Fermi level of the friction material is lower than that of the MHP, electrons transfer from the surface of the MHP film to the former due to the Fermi-level difference, inducing the accumulation of positive charges and forming a localized positive electrostatic field on the surface of the MHP film. The positive electrostatic field interacts with positively charged defects on the surface of the MHP film through the electrostatic repulsion, causing the lattice stretch. The stretched lattice structure slightly widens the band gap and reduces the trap density of the MHP film, which enhances the fluorescence accompanied by a slight blue-shift and prolongs the lifetime of charge carriers, resulting in the TL. In the case of scraping the MHP film with a high-Fermi-level material, a negative electrostatic field is formed, inducing the perovskite lattice compression, which weakens the fluorescence.

## Discussion

We have discovered the TL phenomenon of the MHP film, and revealed an unprecedented TL mechanism based on the friction-induced electron transfer. The TL response is strongly dependent on the relative Fermi level positions of the friction material and the MHP. When the Fermi level of the friction material is lower than that of the MHP, the enhanced fluorescence, namely the TL, can be observed, which is applicable to various types of perovskite materials. Instead, the friction material with a higher Fermi level than that of the MHP reduces the fluorescence of the MHP film. Our results demonstrate that the TL originates from the electron transfer from the MHP to the friction material, forming a positive electrostatic field on the surface of the MHP film, which further leads to the stretched perovskite lattice and thus improved fluorescence and charge carrier lifetime. Our finding uncovers a novel characteristic of the MHP, which can inspire more potential applications of this kind of miraculous materials.

## Materials and methods

All materials were used as received without further purification. MAI, MABr were purchased from Great Cell Solar Materials (Australia). CsBr, FABr were purchased from Yuri Solar Co., Ltd. Glass substrates PbI_2_ and PbBr_2_ were purchased from Advanced Election Technology Co., Ltd. Pb(Ac)_2_·3H_2_O was purchased from TCI chemical Ltd. All solvents were purchased from Sigma-Aldrich without any further purification.

### Preparation of the MHP films

The glass was first cleaned with solvents of ethanol, isopropanol, and ethanol in sequence. Then, the cleaned substrate was treated by the UV-ozone for 30 min. For the MAPbI_3_ film, a 1:3 molar ratio of Pb(Ac)_2_·3H_2_O to MAI was used, with a total Pb^2+^ ion concentration of 0.60 mmol/mL. For the MAPbBr_3_ film, a 1:3 molar ratio of Pb(Ac)_2_·3H_2_O to MABr was used, with a total Pb^2+^ ion concentration of 0.20-1.20 mmol/mL. For the CsPbBr_3_ film, a 1:1 molar ratio of CsBr to PbBr_2_, was used with a total Pb^2+^ ion concentration of 0.20 mmol/mL. For the FAPbBr_3_ film, a 1:1 molar ratio of FABr to PbBr_2_, was used with a total Pb^2+^ ion concentration of 0.50 mmol/mL. MAPbBr_3_ and MAPbI_3_ precursor solution were prepared in DMF. FAPbBr_3_ and CsPbBr_3_ precursor solution were prepared in DMSO. All of these solutions were stirred at room temperature for 8 h. The perovskite films were prepared using a one-step method by spin-coating the precursor solutions on substrate at 6000 r.p.m. for 30 s, followed by annealing at 75 ^o^C for 2 min on hotplate. The triboluminescence can be observed by naked eye under the 365 nm-UV light.

### Characterizations

The XRD and GIXRD analysis were conducted with an X-ray diffraction system (Rigaku) using Cu Kα radiation provided applied current and voltage values of 200 mA and 40 kV, respectively.

Grazing-Incidence Wide-Angle X-Ray Scattering (GIWAXS) data were obtained using the Beamline BL14B1 of the Shanghai Synchrotron Radiation Facility (SSRF) with the incident photon energy of 10 keV (wavelength of 1.2398 Å) at an incident angle of 0.2–0.5° and an exposure time of 30 s.

Ultraviolet photoelectron spectroscopy (UPS) was performed on a photoelectron spectrometer (ESCALAB 250Xi, Thermo Fisher Scientific).

The Kelvin probe force microscopy (KPFM) measurements were performed on a Bruker Dimension VSPM system, which can simultaneously detect the topography and the surface potential of the sample. The equipped KPFM tip was platinum (Pt)-coated Ir probe with ~60 kHz resonance frequency, purchased from Bruker. For the KPFM probe simulation process, the initial surface potential baseline was measured across a 20 μm × 20 μm region of the sample. Subsequent scanning focused on the central 10 μm × 10 μm sub-region, followed by re-mapping the original 20 μm × 20 μm area to document potential changes. Finally, ten repetitive scans were performed within the central 10 μm× 10 μm zone, after which the peripheral 20 μm × 20 μm area was rescanned to quantify the potential evolution.

The steady-state PL spectra and time-resolved PL spectra were recorded on a FLS920 fluorescence spectrometer (Edinburgh Instruments) in air at room temperature. A picosecond pulsed diode laser (406.8 nm) was used as the excitation source. We used photoluminescence (PL)-scanned imaging microscope coupled with a time-correlated single photon counting (TCSPC) module to map the PL kinetics within the perovskite. Excitation of the sample is achieved with a supercontinuum white-light laser (SC400-PP, Fianium, UK) of 450 nm wavelength, 1 MHz repetition rate and ~6 ps pulse width. The laser intensity at samples is adjusted by a neutral density filter and measured with a power meter (PM100D S130VC, Thorlabs, USA). PL image measurement was collected by fast rotation of the galvanometer mirror and using a high-speed detector (HPM-100-50, Hamamatsu, Japan). Each scanning image contains 256 × 256 pixels. The fluorescence signal is collected using a high-speed detector (HPM-100-50, Hamamatsu, Japan).

### DFT calculations

The defect energies and density are calculated using DEC and DDC modules of Defect and Dopant ab initio Simulation Package (DASP) code^47^. A single Γ point is used for Brillouin zone integration for the 192-atom supercell. The defect formation energy of Br vacancies can be calculated as:

$$\Delta {E}_{f}\left({V}_{{Br}}^{q}\right)=E\left({V}_{{Br}}^{q}\right)-E\left({bulk}\right)+E\left({Br}\right)+{\mu }_{{Br}}+q({E}_{F}+{E}_{{VBM}})+{E}_{{corr}}$$

where $E({V}_{{Br}}^{q})$ and E(bulk) refer to the total energy of the supercell with and without a Br vacancy. ${\mu }_{{Br}}$ is the elemental chemical potential of Br referenced to the total energy of elemental Br phase *E(Br)*. ${E}_{F}$ is the Fermi level ranging from 0 to the band gap, and ${E}_{{VBM}}$ is the eigenvalue of the VBM in the defect-free supercell of MAPbBr_3_ at the Γ point. *E*_*corr*_ is the correction for charged defects to account for the finite-size effect^48^. The formation energy of *V*_*Br*_ in enlarged MAPbBr_3_ is calculated following the similar procedure but with a Br vacancy in an enlarged supercell structure. The charge transition level is equal to the Fermi level position, at which the formation energies in two charge states are the same.

Under thermodynamic equilibrium, the density of a defect *V*_*Br*_ in the charge state q is determined by its formation energy according to:

$$n\left({V}_{{Br}}^{q}\right)={N}_{{sites}}{g}_{q}{e}^{(-\Delta {E}_{f}/{k}_{B}T)}$$

where *N*_*sites*_ is the density of the possible defect sites, *g*_*q*_ is the charge-dependent degeneracy factor, *ΔE*_*f*_ is the defect formation energy at the given Fermi level and elemental chemical potentials^49^.

## Supplementary information

Supporting information

Supporting Video S1

## Data Availability

All data are available in the main text or supplementary materials. The data that support the findings of this study are available from the corresponding authors on reasonable request.
