# Supplementary material for: Triboluminescence of metal halide perovskite films
Source: Light Sci Appl. 2025 Nov 6;14:379. doi: 10.1038/s41377-025-02032-4 (PMC12592720; doi:10.1038/s41377-025-02032-4)
Supplement: Supplementary file 1 — Supporting information [file 41377_2025_2032_MOESM1_ESM.docx]

**Supplementary Information for**

**Triboluminescence of Metal Halide Perovskite Films**

Hao Tian,^1,2,#^ Fengke Sun,^2,3,#^ Jun Chen,^1,2,#^ Fusai Sun,^1,2^ Xiaotao Liu,^1^ Yang Li,^1,2^ Zhanjun Zhu,^1,2^ Junxue Guo,^1^ Bao Zhou,^1,2^ Xin Guo,^1,2,*^ Can Li^1,2,*^

^1^ State Key Laboratory of Photoelectric Conversion and Utilization of Solar Energy, Dalian Institute of Chemical Physics, Chinese Academy of Sciences, Dalian 116023, China.

^2^ University of Chinese Academy of Sciences, Beijing 100049, China

^3^ State Key Laboratory of Molecular Reaction Dynamics, Dalian Institute of Chemical Physics, Chinese Academy of Sciences, Dalian 116023, China

^#^ These authors contributed equally to the work

^*^Correspondence to: guoxin@dicp.ac.cn; canli@dicp.ac.cn


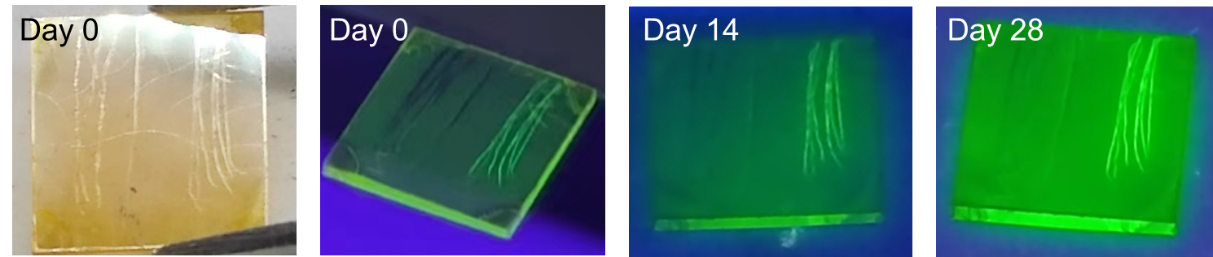


**Supplementary Fig. S1** | Photographs of the MAPbBr_3_ film scraped by Cu and Al at different regions, taken at different days. The left picture was taken under indoor light, while the other three pictures were taken under UV light (365 nm) in glove box.


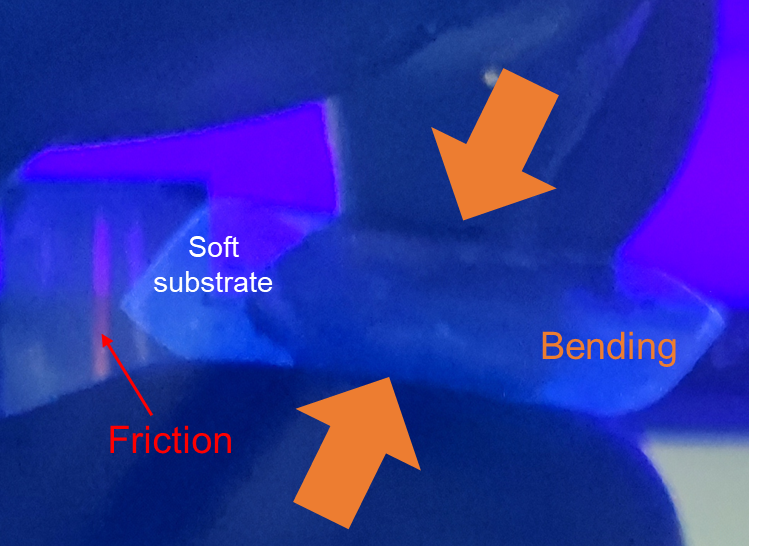


**Supplementary Fig. S2 |** Photograph of the MAPbBr_3_ film on a soft substrate, taken under UV light (365 nm). Bending-induced deformation did not enhance luminescence of the MAPbBr_3_ film.


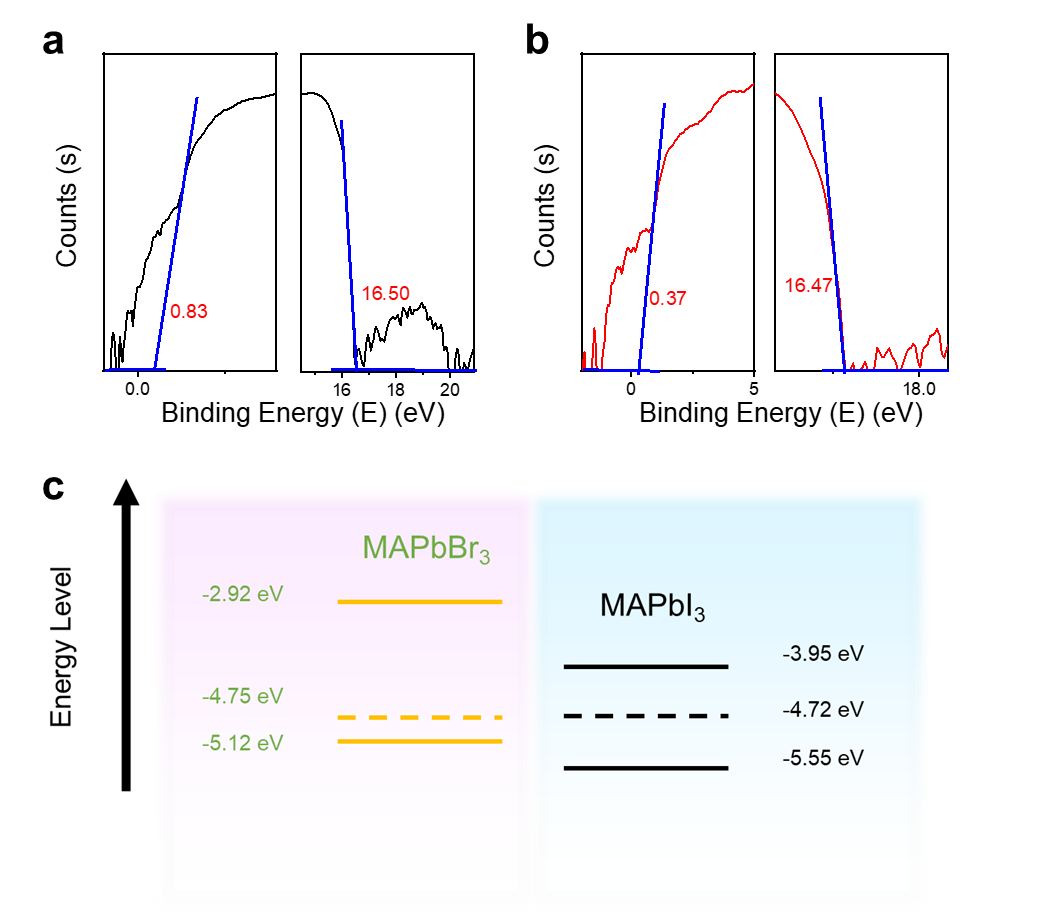


**Supplementary Fig. S3 |** Energy level distribution of MHPs. UPS results of MAPbBr_3_ (a) and MAPbI_3_ (b) films; (c) Schematic diagram of energy levels for MHPs.

**
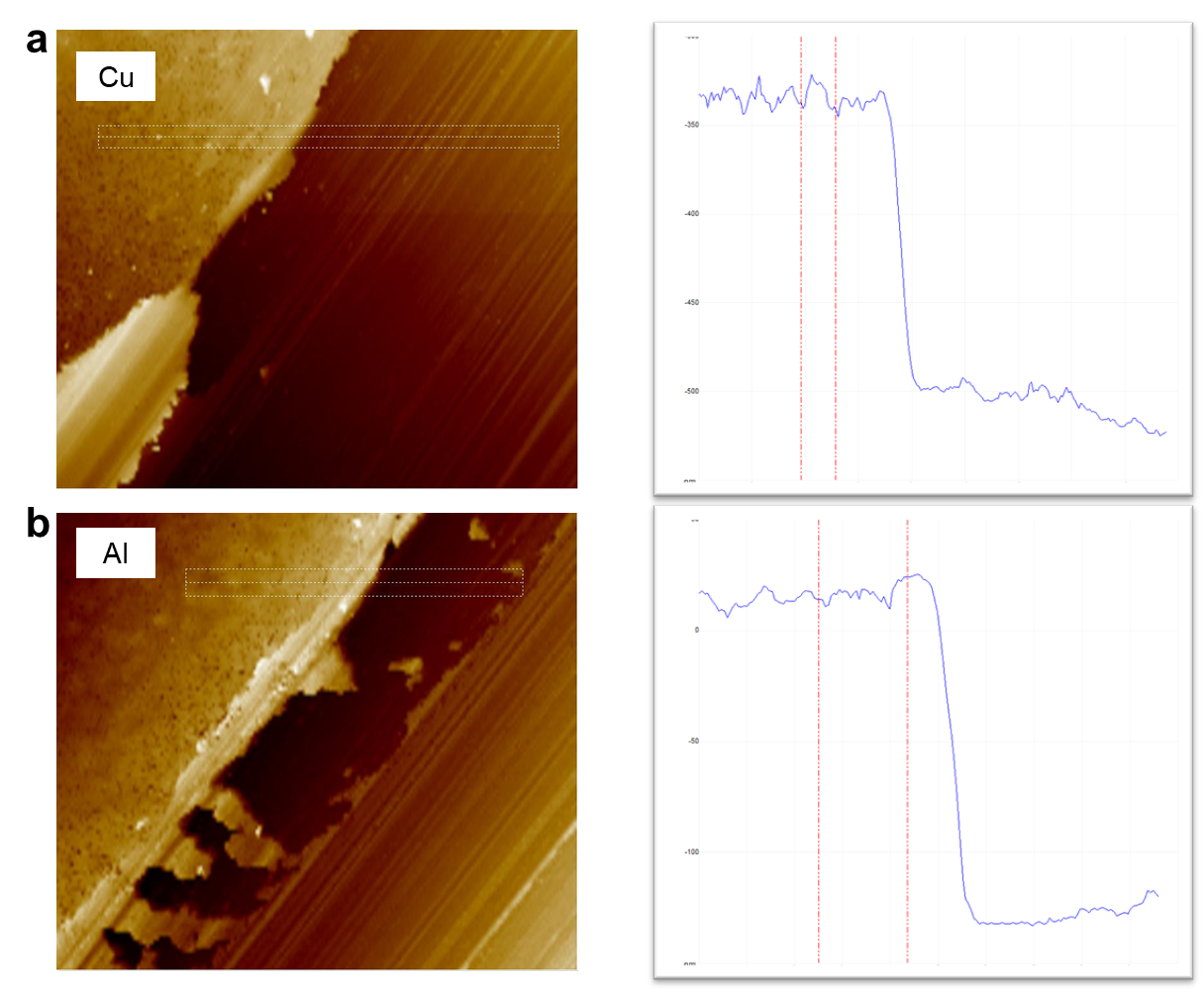
**

**Supplementary Fig. S4 |** AFM results of (a) Cu-friction and (b) Al-friction regions on the MHP film. Each scraping region presents a similar depth of approximately 150 nm, ensuring consistent MHP film thickness after friction with different materials.


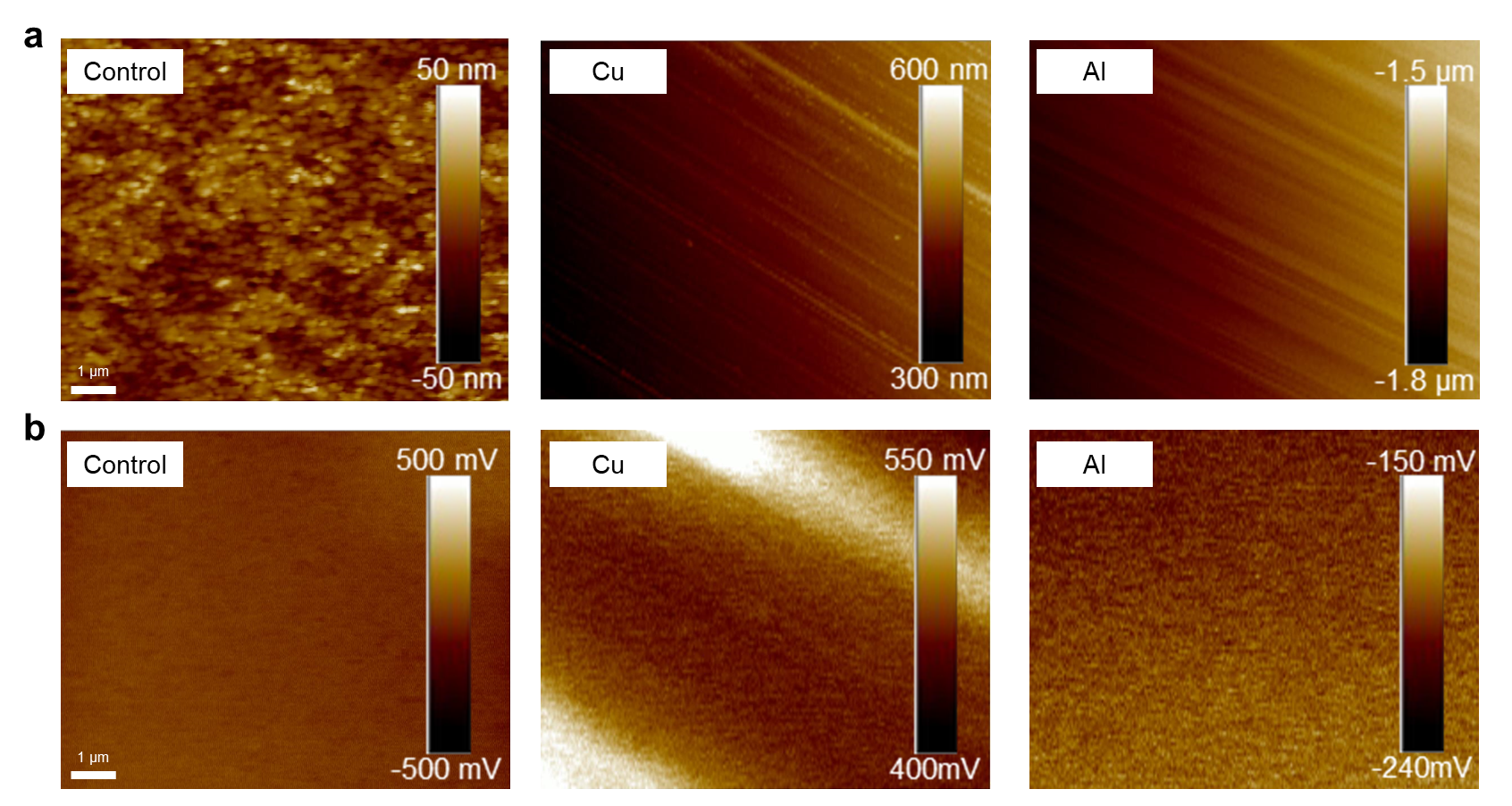


**Supplementary Fig. S5 |** KPFM results of the MHP films. The morphology (a) and surface potential (b) of control, Cu-friction, Al-friction samples, respectively. The perovskite film was divided into three regions; one of them was not scraped as the control, and the other two were scraped by Cu and Al, respectively, with the same friction way.


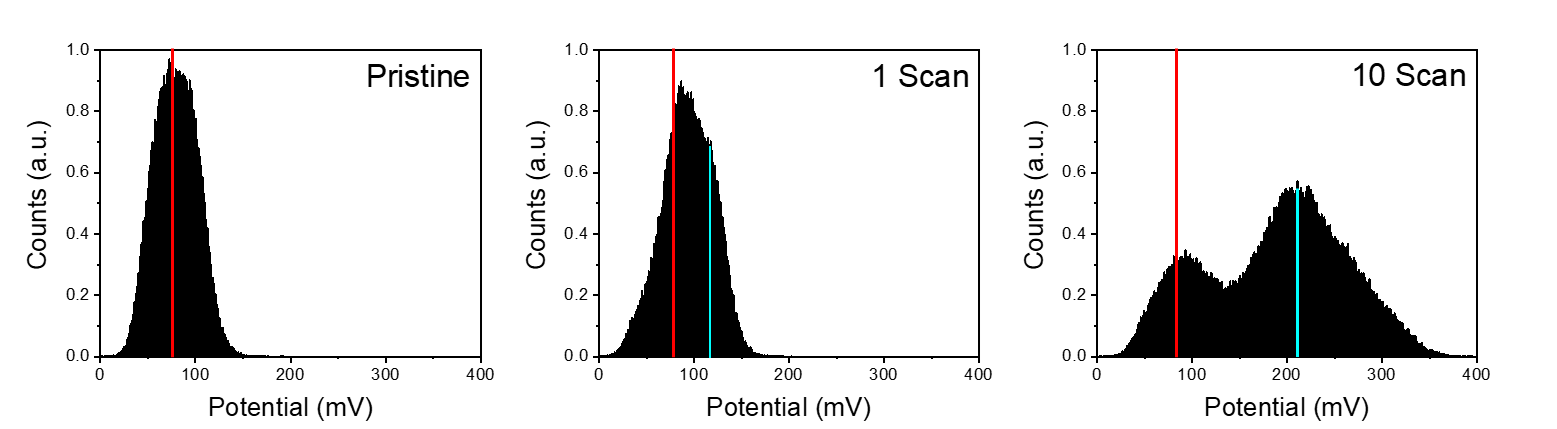


**Supplementary Fig. S6 |** Contact potential difference (CPD) distribution diagram of perovskite films after scanning 0, 1 and 10 times, respectively, using the KPFM probe.

**
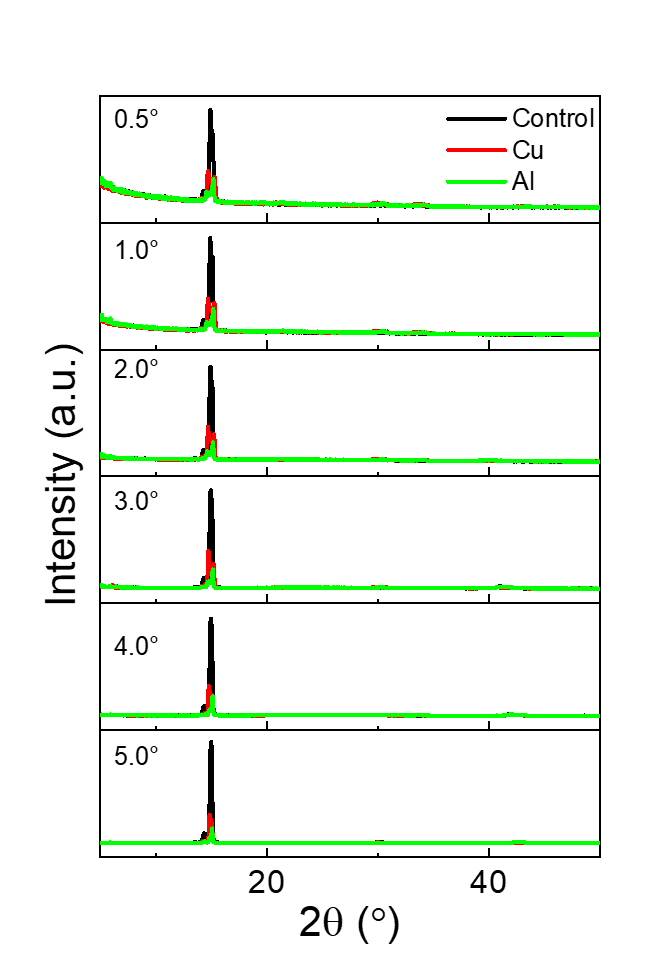
**

**Supplementary Fig. S7 |** GIXRD results (2θ: 5°-50°) of control, Cu-friction and Al-friction samples with different incident angles (0.5-5°). The samples for this measurement were prepared by the same way as that for the KPFM.

**
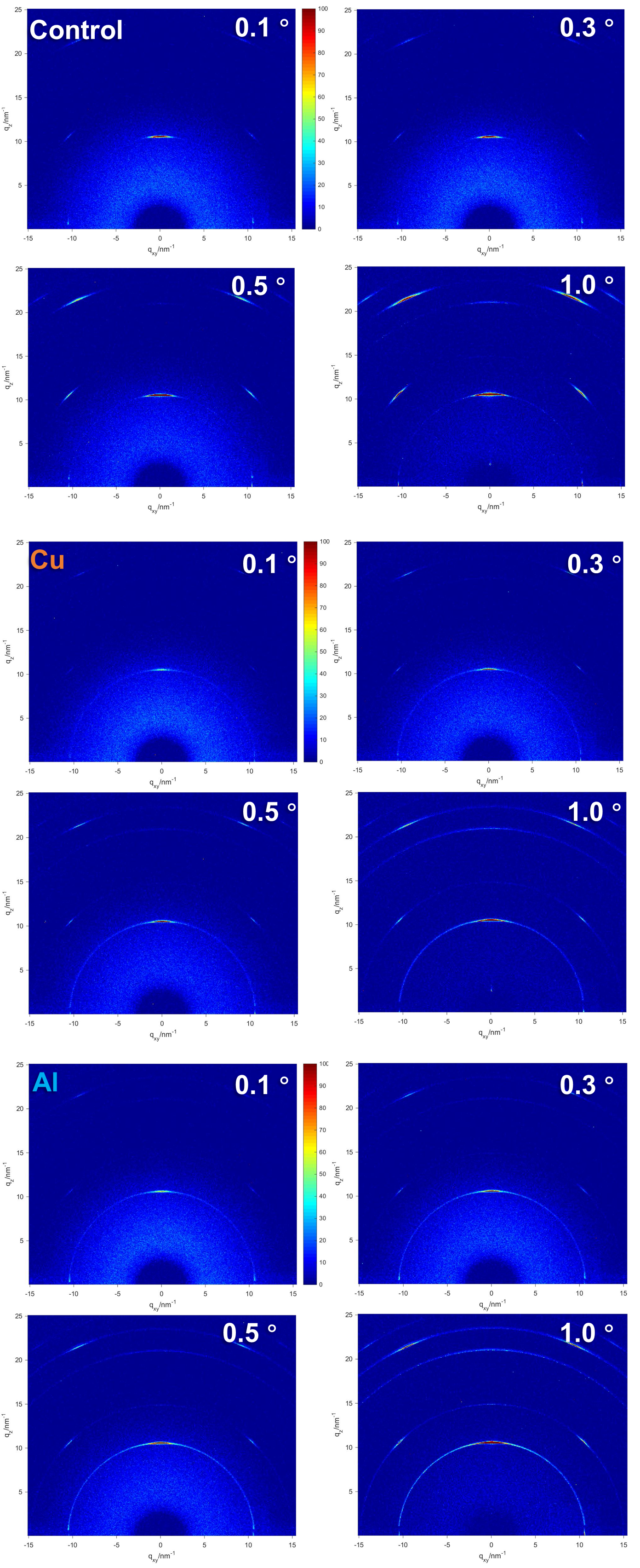
**

**Supplementary Fig. S8 |** Original GIWAXS data of Control, Cu-friction and Al-friction MAPbBr_3_ films with incident angles from 0.1° to 1.0°. The sample production method is the same as the previous test.


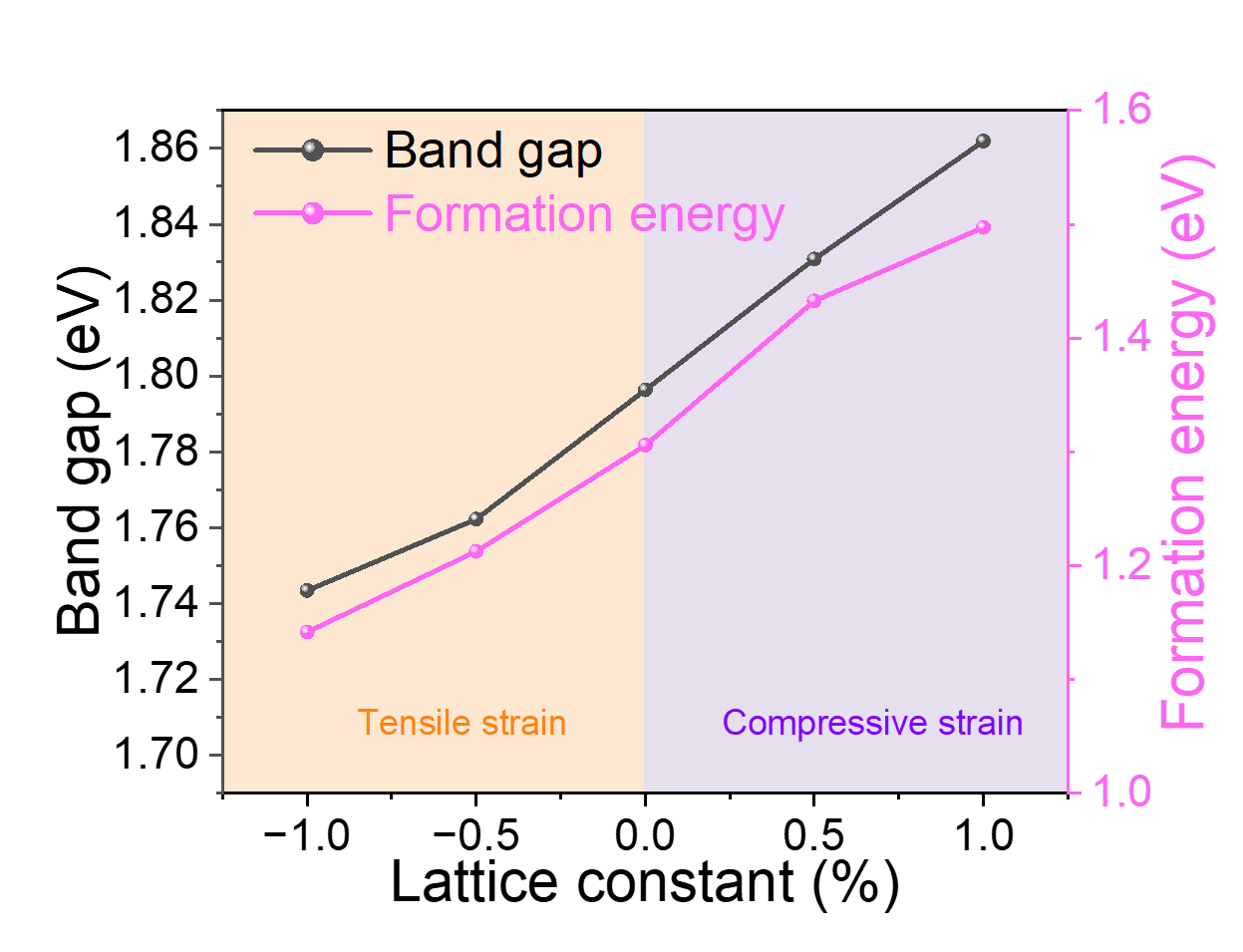


**Supplementary Fig. S9 |** Calculated results of the relationship between the lattice strain (corresponding to the lattice constant) and the bandgap/defect formation energy.

**
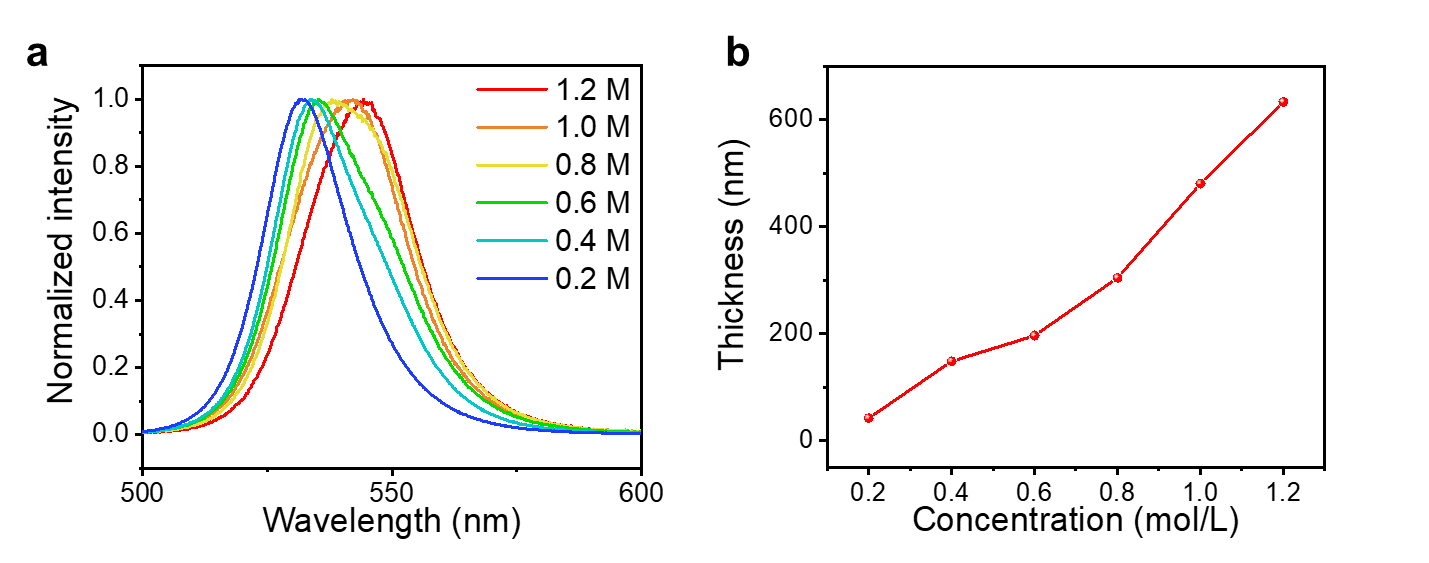
**

**Supplementary Fig. S10** | (a) PL spectra of the MAPbBr_3_ films prepared from precursor solutions with different concentrations. (b) Relationship between thickness and concentration of the MAPbBr_3_ films. These results indicate that the PL spectra of the MAPbBr_3_ film are gradually blue-shifted with the decreased film thickness.

**Supplementary Table S1∣**Energy band parameters based on different lattice constants, obtained from DFT calculations.

| Material | Lattice constant (Å) | Bandgap (eV) | VBM (eV) | CBM (eV) |
| --- | --- | --- | --- | --- |
| MAPbBr_3_ | a=5.97 | 1.7963 | -0.2774 | 1.5189 |
| Stretch | 1.005a | 1.8308 | -0.2877 | 1.5431 |
| Compression | 0.995a | 1.7622 | -0.2712 | 1.4910 |
