# Supplementary material for: Triboluminescence of metal halide perovskite films
Source: Light Sci Appl. 2025 Nov 6;14:379. doi: 10.1038/s41377-025-02032-4 (PMC12592720; doi:10.1038/s41377-025-02032-4)
Supplement: Supplementary file 2 — Supporting Video S1 [file 41377_2025_2032_MOESM2_ESM.docx]

Supplementary Video S1∣The MHP film scraped with high Fermi-level (Al) and low-Fermi level (Cu) materials, respectively.
